# *Pseudomonas coronafaciens* sp. nov., a new phytobacterial species diverse from *Pseudomonas syringae*

**DOI:** 10.1371/journal.pone.0208271

**Published:** 2018-12-06

**Authors:** Bhabesh Dutta, Ronald Gitaitis, Gaurav Agarwal, Teresa Coutinho, David Langston

**Affiliations:** 1 Department of Plant Pathology, Coastal Plain Experiment Station, University of Georgia, Tifton, GA, United States of America; 2 Department of Microbiology and Plant Pathology, Forestry and Agricultural Biotechnology Institute (FABI), University of Pretoria, Pretoria, South Africa; 3 Department of Plant Pathology, Physiology and Weed Science, Tidewater Agricultural Research and Extension Center, Virginia Tech, Suffolk, VA, United States of America; Texas A&M University College Station, UNITED STATES

## Abstract

We propose *Pseudomonas coronafaciens* sp. nov. as a new species in genus *Pseudomonas*, which is diverse from *P*. *syringae*. We also classified strains from onions which are responsible for yellow bud (YB) disease as *P*. *coronafaciens*. Sequencing of 16S rRNA gene and multi-locus sequence analysis (MLSA) of housekeeping genes (*gyrB*, *rpoD*, *gltA* and *gap1* genes) for the *P*. *syringae* pv. *coronafaciens* strains along with other strains of *P*. *syringae* pathovars resulted in a distinct cluster separate from other *P*. *syringae* pathovars. Based on DNA-DNA relatedness, pathotype strain of *P*. *syringae* pv. *coronafaciens* (CFBP 2216^PT^) exhibited ≤35.5% similarity with the pathotype strains of *P*. *syringae* pv. *syringae* (CFBP 1392^PT^, 4702^T^) but exhibited ≥90.6% with the YB strains (YB 12–1, YB 12–4, YB 09–1). Also, the YB strains (YB 12–1, YB 12–4, YB 09–1) were able to infect only onion but not oat, rye and Italian ryegrass (common hosts for *P*. *syrinage* pv. *coronafaciens*). Contrastingly, *P*. *syringae* pv. *coronafaciens* strains (NCPPB 600^PT^, ATCC 19608, Pcf 83–300) produced typical halo blight symptoms on oat, rye and Italian rye grass but did not produce any symptoms on onion. These results provide evidence that *P*. *syringae* pv. *coronafaciens* should be elevated to a species level and the new YB strains may potentially be a novel pathovar of hereto proposed *P*. *coronafaciens* species.

## Introduction

The taxonomy of *Pseudomonas syringae* sensu lato and its pathovars has evolved and been a matter of debate for last 34 years [1]. In the 8^th^ edition of *Bergey’s Manual of Determinative Bacteriology*, *P*. *syringae* was widely accepted as a species and was comprised of fluorescent phytopathogenic *Pseudomonas* nomenspecies [2,3]. Furthermore, a revised taxonomic classification placing 41 nomenspecies of *P*. *syringae* as pathovars, was proposed in the 1^st^ edition of *Bergey’s Manual of Systematic Bacteriology* [4]. This proposal was supported by the International Society for Plant Pathology, subcommittee on taxonomy of plant pathogenic bacteria [5]. The descriptions of most *P*. *syringae* pathovars were based on limited cross-pathogenicity tests on different hosts. As a result, overlap in host-range among different *P*. *syringae* pathovars often occur. Moreover, routine biochemical tests do not differentiate many of the *P*. *syringae* pathovars creating problems in correct identification of pathogens [6,7]. Gardan *et al*. (1999) [8] identified nine ‘genomospecies’ of the *P*. *syringae* complex in a comprehensive DNA-DNA re-association study. Among different genomospecies, genomospecies 4 (also called phylogroup 4) included pathovars of graminaceous species [*P*. *syringae* pv. *coronafaciens* (Elliott) Young et al., *P*. *syringae* pv. *atropurpurea*, *P*. *syringae* pv. *striafaciens*, *P*. *syringae* pv. *oryzae*, and *P*. *syringae* pv. *zizaniae*,], *P*. *syringae* pv. *garcea* [coffee (*Coffea arabica*; Rubiaceae)], and *P*. *syringae* pv. *porri* [leek (*Allium ampeloprasum*; Liliaceae)]. However, genomospecies classifications of *P*. *syrinage* pathovars were not supported by their ribotyping or substrate utilization studies. Detailed polyphasic study using genetic approaches were not conducted. Hence, Gardan *et al*. (1999) [8] refrained from making a formal proposal to elevate *P*. *coronafaciens* to species level and it remained as a pathovar of *P*. *syringae* (*P*. *syringae* pv. *coronafaciens*). Prior to study by Gardan et al. [8], Schaad and Cunfer (1979) [9] tried to differentiate *P*. *syringae* pv. *coronafaciens*, *P*. *syringae* pv. *zea*, *P*. *syringae* pv. *atropurpurea* and *P*. *syringae* pv. *striafaciens*; however, they later concluded that these bacterial species/pathovars are synonymous. These strains did not differ in their physiological, immunological and substrate utilization tests. In addition, little to no differences in their host range was reported as these strains were able to infect oat (*Avena sativa*), rye (*Secale cerale*), wheat (*Triticum aestivum*), barley (*Hordeum vulgare*), smooth bromegrass (*Bromus inermis*), Japanese brome (*B*. *japonicas*), chess brome (*B*. *secalinus*), cheatgrass (B. tectorum), quackgrass (*Agropyron repens*), maize (*Zea mays*).

In this paper, we propose the elevation of *P*. *syringae* pv. *coronafaciens* to a species level as *P*. *coronafaciens*, which was confirmed by various molecular and biochemical methods including sequencing of the 16S rRNA gene, and multi-locus sequence analysis (MLSA) based on sequences of housekeeping genes *gyrB*, *rpoD*, *gltA*, and *gap1*, substrate utilization tests (BIOLOG), polymerase chain reaction (PCR) analysis using plasmid (pCOR1), coronafactate ligase (cfl) and *HrpZ* effectors genes-specific primers, and DNA-DNA-hybridization. We also characterized strains from onion which are responsible for yellow bud (YB) disease [10] and concluded that they may potentially be a novel pathovar of hereto proposed *P*. *coronafaciens* species.

## Materials and methods

### Bacterial strains used in this study

Bacterial strains used in this study included *P*. *syringae* pv. *coronafaciens* NCPPB 600^PT^ = CFBP 2216 ^PT^ (pathotype strain) and ATCC 19608, *P*. *syringae* pv. *syringae* NCPPB 281 ^PT^ = CFBP 4702 ^PT^ (pathotype strain) and NCPPB 1770, and *P*. *syringae* pv. *aptata* NCPPB 3539, *P*. *coronafaciens* pv. *garcea* NCPPB 588 ^PT^ (pathotype strain), *P*. *coronafaciens* pv. *oryzae* NCPPB 3683 ^PT^ (pathotype strain), *P*. *coronafaciens* pv. *porri* NCPPB 3364 ^PT^ (pathotype strain), *P*. *coronafaciens* pv. *striafaciens* NCPPB 1898 ^PT^ (pathotype strain), *P*. *cannabina* NCPPB 1437, and *P*. *savastanoi* NCPPB 639 ^PT^ (pathotype strain). The strains were recovered following instructions given by the NCPPB and ATCC culture collections. Other bacterial strains used in this study are listed in Table 1. The YB strains from onion were maintained on nutrient agar (NA) supplemented with 0.5% yeast extract (NA+).

**Table 1:** List of bacterial strains used in this study.

Species ID	Strain	Host	Strain source
*Pseudomonas cannabina*	ICMP 4326	Radish	GenBank
*P*. *syringae* pv. *coronafaciens*	NCPPB 600 ^PT^ = CFBP 2216^PT^	Oat	NCPPB^a^, UK; CFBP^b^, France
	ATCC 19608	Oat	ATCC^c^, U.S.A.
	Pcf 93–2	Oat	CPES^d^, UGA
	Pcf 83–300	Rye	CPES, UGA
	Pcf 83–302	Oat	CPES, UGA
	YB 12–1	Onion	CPES, UGA
	YB 09–1	Onion	CPES, UGA
	YB 12–4	Onion	CPES, UGA
	YB 12–5	Onion	CPES, UGA
*P*. *syringae* pv. *garcea*	NCPPB 588 ^PT^	Coffee	NCPPB, UK
*P*. *syringae* pv. *oryzae*	NCPPB 3683 ^PT^	Rice	NCPPB, UK
*P*. *syringae* pv. *porri*	NCPPB 3364 ^PT^	Leak	NCPPB, UK
*P*. *syringae* pv. *striafaciens*	NCPPB 1898 ^PT^	Oat	NCPPB, UK
*P*. *putida*	ATCC 12633	-	GenBank
*P*. *savastanoi*	NCPPB 639^T^	Olive	GenBank
*P*. *syringae* pv. *aptata*	NCPPB 3539	Sugarbeet	NCPPB, UK
*P*. *syringae* pv. *glycinea*	Psg 86–3	Soybean	CPES, UGA
*P*. *syringae* pv. *lachrymans*	Psl 83–1	Cucumber	CPES, UGA
*P*. *syringae* pv. *morsprunorum*	Psm 83–4	Peach	CPES, UGA
*P*. *syringae* pv.*phaseolicola*	Pph 83–2	Kudzu	CPES, UGA
*P*. *syringae* pv. *syringae*	NCPPB 281^PT^ = CFBP 4702^PT^	Lilac	NCPPB, UK; CFBP, France
	NCPPB 1770	Bean	NCPPB, UK
	CFBP 1392	Lilac	CFBP, France
	Pss 87–300	Bean	CPES, UGA
	Pss 88–306	Bean	CPES, UGA
*P*. *syringae* pv. *tomato*	Pst 84–17	Tomato	CPES, UGA
	Pst 89–21	Tomato	CPES, UGA
*P*. *viridiflava*	CFBP 2107^T^ = ATCC 13223^T^	Bean	GenBank

^**P**T^Pathotype strain.

^a^NCPPB = National Culture Collection for Plant Pathogenic Bacteria, Sandhutton, York, UK.

^b^CFBP = Collection Française de Bactéries Associées aux Plantes, Beaucouze Cedex, France.

^c^ATTC = American Type Culture Collection, Manassas, VA, USA.

^d^Coastal Plain Research Station, University of Georgia, Tifton, GA, USA.

### Phylogenetic analysis based on 16S rRNA, *gap1*, *gyrB*, *gltA* and *rpoD* gene sequences

Total microbial genomic DNA from bacterial strains was extracted using an UltraClean Microbial DNA Kit (MO BIO, Carlsbad, CA) according to the manufacturer’s instructions. Two microliters of bacterial DNA (1 ng/ μl) were amplified in 25 μl of a PCR master mix using the 16S rRNA primer pair (fD1 AGAGTTTGATCCTGGCTCAG and rD1 AAGGAGGTGATCCAGCC) as described by Weisburg et al. (1991) [11]. For sequencing, the PCR amplicon from these bacterial strains were purified using an affinity column (Wizard PCR Preps DNA Purification System, Promega) and sequenced (Eurofins Genomics, Huntsville, AL, USA). ClustalW [12] was used for sequence alignment and overhangs were trimmed. PAUP*4.0b.10 [13] was used for phylogenetic analyses. Phylogenetic trees were created using parsimony analysis. Bootstrap analysis (10,000 replications) was performed for the parsimony tree using stepwise addition with the tree-bisection reconnection (TBR) branch-swapping option. As an outgroup, *P*. *putida* was included in the analysis.

Purified DNA from four YB strains (12–1, 09–1, 12–4, 12–5) along with pathotype strains of *P*. *syringae* pv. *syringae*, *P*. *syringae* pv. *coronafaciens*, *P*. *syringae* pv. *garcea*, *P*. *syringae* pv. *oryzae*, *P*. *syringae* pv. *striafacien*s, and *P*. *syringae* pv. *porri* (NCPPB 3539) were extracted using an UltraClean Microbial DNA Kit (MO BIO, Carlsbad, CA) according to the manufacturer’s instructions. DNA extraction from additional *P*. *syringae* pathovars (*P*. *syringae* pv. *tomato*, *P*. *syringae* pv. *maculicola*, *P*. *syringae* pv. *lachrymans*, *P*. *syringae* pv. *glycinea*, *P*. *syringae* pv. *morsprunorum*, and *P*. *syringae* pv. *phaseolicola*) strains was also conducted. Four housekeeping genes (*gap1*, *gltA*, *gyrB*, and *rpoD*) were amplified for each bacterial strain stated above with primers described by Hwang et al. (2005) [14] and PCR products were sequenced as described by Yan et al. (2008) [15]. Sequence analysis and tree construction were performed on concatenated sequences as described above.

### PCR assay for the detection of plasmid pCOR1 and coronafactate ligase gene

The detection of pCOR1 plasmid in YB strains were conducted as per the conventional PCR protocol described by Takahashi et al. (1996) [16] using primer pairs P1(5’ GGGCTGCAGGAGAGTCCCAATGGA-3’) and P2 (5’-TTCCTGCAGAGCTATGGCCACTTG-3’). Four YB strains (12–1, 09–1, 12–4, 12–5) along with a pathotype strain (NCPPB 600^PT^) and a strain (ATCC 19608) of *P*. *coronafaciens*, a pathotype strain (NCPPB 281 ^PT^) and a strain (NCPPB 1770) of *P*. *syringae* pv. *syringae* and one strain each of *P*. *syringae* pv. *phaseolicola*, *P*. *syringae* pv. *glycinea*, *P*. *syringae* pv. *lachrymans*, *P*. *syringae* pv. *maculicola*, *P*. *syringae* pv. *tomato*, *P*. *syringae* pv. *syringae*, and *P*. *syringae* pv. *aptata* were used in this assay. The gene, coronafactate ligase encodes the coupling of coronafacic acid and coronamic acid in a coronatine (phyotoxin) biosynthetic pathway [1]. For coronafactate ligase PCR, microbial DNA were amplified using a primer pair, CFL F 5’-GGCGCTCCCTCGCACTT-3’ and CFL R 5’-GGTATTGGCGGGGGTGC-3’ following conditions described by Bereswill et al. (1991) [17]. The PCR detection assay for the *cfl* gene was conducted with the strains described above.

### The gene, *hrpZ* based PCR assay

The *hrpZ* based PCR assays with group I-IV specific primers were conducted for the YB strains (12–1, 09–1, 12–4, 12–5) as described previously by Inoue and Takikawa (2006) [18]. Known strains of *P*. *syringae* pv. *phaseolicola*, *P*. *syringae* pv. *lachrymans*, *P*. *syringae* pv. *tomato*, *P*. *syringae* pv. *syringae*, and *P*. *syringae* pv. *coronafaciens* (NCPPB 600 ^PT^ and ATCC 19608) were used as positive controls for *hrpZ* group Ia, Ib, II, III, and IV-based PCR assays, respectively.

### PCR assay for the detection of effector genes (*avrPto*, *avrD1*, *avrAE1*, *hopA1*, *hopB1*, *hopC1*, *hopD1*, *hopF2*, *hopG1*, *hrpK1*, *hopAF1*, and *hopAN1*)

The presence of 12 effector genes (*avrPto*, *avrD1*, *avrAE1*, *hopA1*, *hopB1*, *hopC1*, *hopD1*, *hopF2*, *hopG1*, *hrpK1*, *hopAF1*, and *hopAN1*) were assayed by PCR amplification using specific primers under conditions described by Ferrante and Scortichini [19]. Two YB strains (12–1, 09–1 and 12–4) and the strains of *P*. *syringae* pv. *syringae* (NCPPB 281 ^PT^ and NCPPB 1770) and *P*. *syringae* pv. *coronafaciens* (NCPPB 600 ^PT^ and ATCC 19608) were used.

### DNA-DNA hybridization and determination of DNA G+C content

High-quality DNA for DNA–DNA hybridization was prepared by the method of Wilson (1987), with minor modifications [20,21]. DNA–DNA hybridization was performed using the microplate method with some modifications [20, 21]. The hybridization temperature was 45±1°C. The strains were labeled with 4-methylumbelliferyl-beta-D-galactoside and the fluorescence intensity was measured. Reciprocal reactions were performed for select hybridization pairs and variation within the limits of this method [22]. The DNA G+C contents for the pathotype strain of *P*. *syringae* pv. *coronafaciens* (NCPPB 600 ^PT^) and an onion strain (YB 12–1) was measured by HPLC [23,24].

### Phenotypic characteristics

Physiological and biochemical tests were performed on the pathotype strain of *P*. *syringae* pv. *coronafaciens* strains (NCPPB 600^PT^) along with onion strains (12–1, 09–1, 12–4, 12–5), rye strain (*P*. *syringae* pv. *coronafaciens* 83–300), and oat strain (*P*. *syringae* pv. *coronafaciens* 93–2). Results were compared with *P*. *syringae* pv. *syringae* strains (NCPPB 1770, *P*. *syringae* pv. *syringae* 87–300, *P*. *syringae* pv. *syringae* 88–306). The phenotypic characteristics of additional *P*. *syringae* pathovar strains (*P*. *syringae* pv. *aptata*, *P*. *syringae* pv. *glycinea*, *P*. *syringae* pv. *lachrymans*, *P*. *syringae* pv. *maculicola*, *P*. *syringae* pv. *morsprunorum*, *P*. *syringae* pv. *phaseolicola*, and *P*. *syringae* pv. *tomato*) were adopted from the literature [25] for comparison. BIOLOG GN2 plates were used to test substrate utilization patterns for the strains characterized. Additional tests included utilization of trigonelline, mannitol, erythritol, sorbitol, inositol, D-tartarate, L-lactate, ability to reduce nitrate to nitrite, ability to form pits on crystal violet pectate (pectinolytic) and carboxymethy cellulose media (cellulolytic), ability to hydrolyze starch, esculin and gelatin, indole reaction, LOPAT test (levan production, oxidase activity, pectinolytic (potato rot) activity, arginine dihydrolase, tobacco hypersensitivity), fluorescence on King’s B medium (KMB) and ice-nucleation activity tests [25].

### Fatty acid analysis

The whole-cell fatty acid methyl ester (FAME) composition was determined for the type strain of *P*. *coronafaciens* (NCPPB 600 ^PT^) and an onion strain (YB 12–1). Strains were cultured on tryptic soy broth agar for 24 h at 28°C, and whole-cell fatty acids were saponified, methylated, and extracted as described previously by Miller and Berger (1985) [26]. FAME analysis was conducted using the Microbial Identification System, Sherlock version 3.10 (MIDI).

### Pathogenicity test

Three YB strains (12–1, 09–1, 12–4) and *P*. *syringae* pv. *coronafaciens* (NCPPB 600 ^PT^, ATCC 19608, Pcf 83–300), *P*. *syringae* pv. *porri* (NCPPB 3364 ^PT^) and *P*. *syringae* pv. *syringae* (NCPPB 281 ^PT^, Pss 87–300) were grown overnight at 28°C in nutrient broth on a rotary shaker (Innova; New Brunswick Scientific Co., Edison, NJ) at 150 rpm. After overnight incubation, bacterial cultures were centrifuged at 5,000 × g (Allegra 25R, Beckman Coulter, Fullerton, CA) for 3 min and the supernatant was decanted leaving a pellet of bacterial cells. The pellet was resuspended in 0.1 M PBS and the concentration of each bacterial strain was adjusted using a spectrophotometer (Spectronic 20, Bausch and Lomb, Rochester, NY) to an optical density of 0.3 at 600 nm (≈1 × 10^8^ CFU/ml). Seedlings of rye (cv. Wren Abruzzi), oat (cv. Gerard 229), Italian ryegrass (cv. Attain) and onion seedlings (cv. Century) were planted in 10 cm × 8 cm (diameter × height) pots (Hummert International, Earth City, MO) in a commercial potting mix (Sunshine LP5 Plug Mix; Sun Gro Horticulture Industries, Bellevue, WA) in the greenhouse and maintained at 22–24°C and 70–75% RH with a 12L:12D photoperiod. Three weeks-old seedlings (*n* = 10/strain/experiment) of each host type were inoculated using a hypodermic syringe and needle to inject a 1.0 ml suspension containing 1 × 10^8^ CFU/ml of each bacterial strain in the leaf. Seedlings inoculated with PBS served as a negative control. Inoculated seedlings were evaluated for development of symptoms up to 15 days post inoculation (DPI).

## Results

### Phylogenetic analysis based on 16S rRNA, *gap1*, *gyrB*, *gltA* and *rpoD* gene sequences

**Fig 1:**
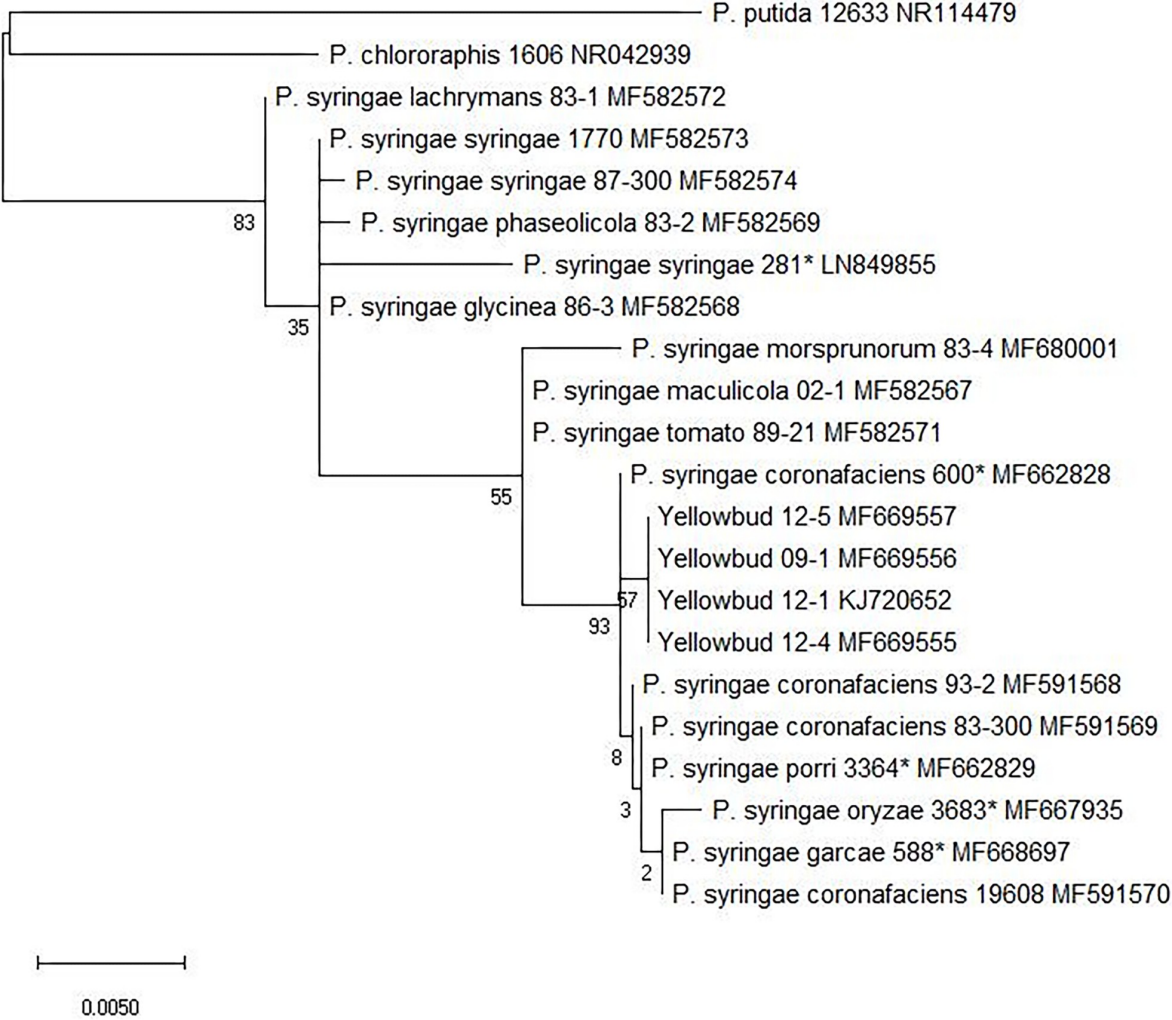
Maximum parsimony tree based on nucleotide sequences of the 16S rRNA gene of *Pseudomonas* species and pathovars obtained from heuristic parsimony search and bootstrap analysis. Bootstrap values are shown at the nodes based on 10,000 replications. Gaps were treated as missing data. The 16S rRNA gene sequence of *Pseudomonas putida* obtained from NCBI database was treated as an outgroup. Bar, 0.001 substitutions per nucleotide position. The accession numbers are listed adjacent to the respective bacterial strain. The “*” in the figure represents a pathotype strain.

Based on 16S rRNA gene sequences, strains of *P*. *syringae* pv. *coronafaciens* [NCPPB 600 ^PT^, ATCC 19608, 93–2, 83–300] and YB from onion (09–1, 12–1, 12–4, and 12–5), and the pathotype strains of *P*. *syringae* pv. porri (NCPPB 3364 ^PT^), *P*.*syringae* pv. *oryzae* (NCPPB 3683^PT^), and *P*.*syringae* pv. *garcea* (NCPPB 588^PT^) formed a clade that was distinct from other *P*. *syringae* pathovars (Fig 1).

**Fig 2:**
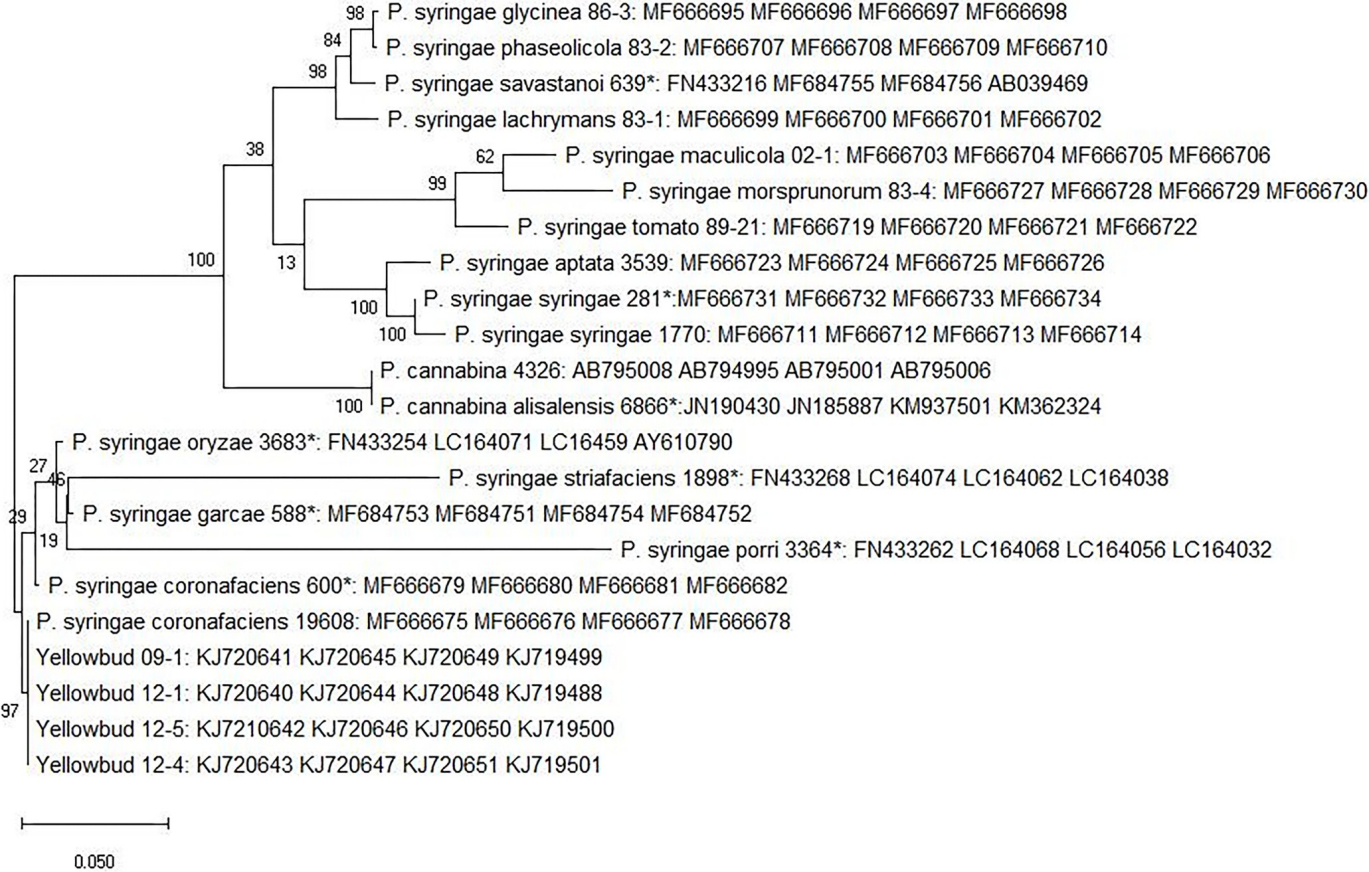
Maximum parsimony tree based on nucleotide sequences of housekeeping genes *gltA*, *gap1*, *gyrB*, and *rpoD* of *Pseudomonas* species and pathovars obtained from heuristic parsimony search and bootstrap analysis. Bootstrap values are shown at the nodes based on 10,000 replications. Gaps were treated as missing data. Bar, 0.01 substitutions per nucleotide position. The accession numbers in the figure are in following order for each strain: *rpoD*, *gap1*, *gltA* and *gyrB*. The “*” in the figure represents a pathotype strain.

Sequences of the four housekeeping gene loci *gltA*, *gap1*, *gyrB*, and *rpoD* [15] were concatenated for the strains described above (Fig 2). The four YB strains (12–1, 09–1, 12–4, 12–5) along with the pathotype strain of *P*. *syringae* pv. *coronafaciens* (NCPPB 600 ^PT^) and a strain (ATCC 19608) formed a distinct clade. This clade also contained the pathotype strains of *P*. *syringae* pv. porri (NCPPB 3364 ^PT^), *P*.*syringae* pv. *oryzae* (NCPPB 3683^PT^), *P*.*syringae* pv. *striafaciens* (NCPPB 1898^PT^) and *P*.*syringae* pv. *garcea* (NCPPB 588^PT^) separate from other *P*. *syringae* pathovars (Fig 2). These results suggest that strains from *P*. *syringae* pv. *coronafacien*s clade are closely related and different from other *P*. *syringae* pathovars.

### PCR assay for the detection of plasmid pCOR1 and coronafactate ligase (*cfl*) gene

As expected, PCR amplification of both pathotype (NCPPB 600 ^PT^) and a strain of *P*. *syringae* pv. *coronafaciens* (ATCC 19608) with pCOR1-specific primers resulted in a 600 base pair (bp) amplicon (Table 2). One hundred percent of the YB strains (12–1, 09–1, 12–4, 12–5) also produced an amplicon of same size indicating a presence of indigenous plasmid (pCOR1) which is responsible for biosynthesis of coronatine toxin. None of the bacterial strains of *P*. *syringae* pathovars including strains of *P*. *syringae* pv. *syringae* (NCPPB 281 ^PT^) and *P*. *syringae* pv. *aptata* (NCPPB 3539 ^PT^) were amplified by the pCOR1-based PCR assay (Table 2). These results also suggest that the YB strains are closely related to *P*. *syringae* pv. *coronafaciens* and both are different from the *P*. *syringae* pathovars tested. The *cfl* gene was detected from all four YB strains (12–1, 09–1, 12–4, 12–5), *P*. *coronafaciens* (NCPPB 600 ^PT^ and ATCC 19608), *P*. *syringae* pv. *glycinea*, *P*. *syringae* pv. *maculicola*, and *P*. *syringae* pv. *tomato* with an expected amplicon size of 600 bp (Table 2). This gene was not detected from other *P*. *syringae* pathovars (*P*. *syringae* pv. *phaseolicola*, *P*. *syringae* pv. *lachrymans*, *P*. *syringae* pv. *syringae*, and *P*. *syringae* pv. *aptata*) tested (Table 2).

**Table 2:** Polymerase chain reaction screening of bacterial strains using of plasmid (pCOR1)- and coronafacate ligase (*cfl*) gene-specific primers.

Species or pathovars of *Pseudomonas*	Strain	pCOR1	*cfl*
*P*. *syringae* pv. *phaseolicola*	83–2	-^a^	-
*P*. *syringae* pv. *glycinea*	86–3	-	+^b^
*P*. *syringae* pv. *lachrymans*	83–1	-	-
*P*. *syringae* pv. *maculicola*	02–1	-	+
*P*. *syringae* pv. *tomato*	84–17, 89–21	-	+
*P*. *syringae* pv. *syringae*	NCPPB 1770 NCPPB 281 ^PT^	-	-
*P*. *syringae* pv. *aptata*	NCPPB 3539	-	-
*P*. *coronafaciens*	ATCC 19608, NCPPB 600 ^PT^ YB 12–1, YB 09–1, YB12-4, YB 12–5	+	+

^a^Negative detection by polymerase chain reaction.

^b^Positive detection by polymerase chain reaction.

^PT^Pathotype strain.

### The gene, *hrpZ* based PCR assay

Only YB strains (12–1, 09–1, 12–4, 12–5) and *P*. *syringae* pv. *coronafaciens* strains (NCPPB 600 ^PT^ and ATCC 19608) were amplified with *hrpZ* group IV based PCR primers and produced an amplicon of 780 bp (Table 3). In contrast, amplicons were not produced by any of the *P*. *syringae* pv. *coronafaciens* strains, including the YB strains with *hrpZ* group Ia-III-based PCR assays. *P*. *syringae* pv. *phaseolicola*, *P*. *syringae* pv. *lachrymans*, *P*. *syringae* pv. *tomato*, and *P*. *syringae* pv. *syringae*, were only amplified by PCR assays with *hrpZ* group Ia (880 bp), Ib (850 bp), II (1000 bp), and III (750 bp) primers, respectively (Table 3). These results indicate that the YB strains belong to *hrpZ* group IV have a close relationship with *P*. *syringae* pv. *coronafaciens* strains. These results also indicate that *P*. *syringae* pv. *coronafaciens* strains group differently from *P*. *syringae* pathovars.

**Table 3:** Polymerase chain reaction screening of bacterial strains using *hrpZ* Group I-IV specific primers.

Species or pathovars of *Pseudomonas*	Strain	*HrpZ* groups				
		*hrpZ* G-IA	*hrpZ* G-IB	*hrpZ* G-II	*hrpZ* G-III	*hrpZ* G- IV
*P*. *syringae* pv. *phaseolicola*	83–2	+	-	-	-	-
*P*. *syringae* pv. *glycinea*	86–3	+	-	-	-	-
*P*. *syringae* pv. *lachrymans*	83–1	-^a^	+^b^	-	-	-
*P*. *syringae* pv. *maculicola*	02–1	-	+	-	-	-
*P*. *syringae* pv. *tomato*	84–17, 89–21	-	-	+	-	-
*P*. *syringae* pv. *syringae*	NCPPB 1770 NCPPB 281 ^PT^	-	-	-	+	-
*P*. *syringae* pv. *aptata*	NCPPB 3539	-	-	-	+	-
*P*. *coronafaciens*	ATCC 19608 NCPPB 600^PT^ YB 12–1, YB 09–1, YB 12–4, YB 12–5	-	-	-	-	+

^a^Negative detection by polymerase chain reaction.

^b^Positive detection by polymerase chain reaction.

^PT^Pathotype strain.

### PCR assay for the detection of effector genes (*avrPto*, *avrD1*, *avrAE1*, *hopA1*, *hopB1*, *hopC1*, *hopD1*, *hopF2*, *hopG1*, *hrpK1*, *hopAF1*, and *hopAN1*)

Both YB and *P*. *syringae* pv. *coronafaciens* strains were positive by PCR assay for *avrPto*, *avrD1*, *avrAE1*, *hopA1*, *hopB1*, *hopD1*, and *hopAF1* genes. These bacterial strains were negative for *hopC1*, *hopF2*, *hopG1*, *hrpK1*, and *hopAN1* genes (Table 4). *P*. *syringae* pv. *syringae* was also positive for the effector genes (*avrPto*, *avrD1*, *avrAE1*, *hopC1*, and *hopAN1*) whereas it was negative for *hopA1*, *hopB1*, *hopD1*, *hopF2*, *hopG1*, *hrpK1*, and *hopAF1* genes (Table 4).

**Table 4:** List of effector proteins and aviruluence genes screened by polymerase chain reaction for the bacterial strains of *Pseudomonas coronafaciens* and *Pseudomonas syringae* pv. *syringae*.

Bacterial Strains^a^		
Effector Genes	*Pseudomonas syringae pv*. *coronafaciens*	*P*. *syringae* pv. *syringae*
avrPto	+ ^b^	+
avrD1	+	+
avrAE1	+	+
hopA1	+	-^c^
hopB1	+	-
hopC1	-	+
hopD1	+	-
hopF2	-	-
hopG1	-	-
hrpK1	-	-
hopAF1	+	-
hopAN1	-	+

^a^*Pseudomonas coronafaciens* Strains: YB 12–1, YB 09–1, YB 12–4, ATCC 19608 and NCPPB 600 ^PT^; *P*. *syringae* pv. *syringae* NCPPB 281 ^PT^.

^b^Positive detection by polymerase chain reaction.

^c^Negative detection by polymerase chain reaction.

^PT^Pathotype strain.

### DNA-DNA hybridization and determination of DNA G+C content

Three representative YB strains (09–1, 12–1 and 12–4) exhibited high levels of DNA–DNA relatedness to each other (≥94.9%) (Table 5). In contrast, DNA-DNA relatedness of the three YB strains with a pathotype strain of *P*. *syringae* pv. *syringae* (CFBP 4702^PT^ = NCPPB 281^PT^) displayed ≤40.8% similarity (Table 5). Additionally, the type strain of *P*. *syringae* pv. *coronafaciens* (CFBP 2216^PT^ = NCPPB 600^PT^) exhibited 34.2% similarity with the type strain of *P*. *syringae* pv. *syringae* (CFBP 4702^PT^ = NCPPB 281^PT^). Furthermore, DNA-DNA relatedness of a representative strain of YB (12–1) when compared with the pathotype strains of *P*. *syringae* pv. *coronafaciens* (CFBP 2216^PT^) and *P*. *syringae* pv. *syringae* (4702), exhibited 90.6% and 36.2% similarity, respectively (Table 5). The DNA G+C% for NCPPB 600 ^PT^ and YB 12–1 was 58.2 and 57.8 mol%, respectively.

**Table 5:** DNA relatedness among *Pseudomonas coronafaciens* and *P*. *syringae* pv. *syringae*.

Source of unlabeled DNA		Source of ^3^H-labeled DNA					
Taxon	Strain	CFBP 2216	YB 12–4	YB 09–1	YB 12–1	CFBP 1392	CFBP 4702
*P*. *syringae pv*. *coronafaciens*	CFBP 2216 ^PT^ = NCPPB^a^ 600^PT^	100^b^	*	*	90.6	35.5	34.2
"	YB 12–4	*^c^	*	*	*	*	*
"	YB 09–1	*	99.3	100	94.9	*	40.8
"	YB 12–1	90.6	100	*	100	*	37.8
*P*. *syringae* pv. *syringae*	CFBP 1392	*	*	*	34.8	100	100
"	CFBP 4702^PT^ = NCPPB 281^PT^	*	*	*	36.2	*	100

^a^Strain designations are according to CFBP = Collection Française de Bactéries Associées aux Plantes, Beaucouze Cedex, France and NCPPB = National Culture Collection for Plant Pathogenic Bacteria, Sandhutton, York, UK

^b^ The values in the table represent DNA relatedness, expressed as percentage relative re-association of the particular combination of DNA isolated from different strains.

^c^Represent strains not compared.

^PT^Pathotype strain.

### Phenotypic characteristics

The most useful phenotypic characteristics for the differentiation of the *P*. *syringae* pv. *coronafaciens* strains from *P*. *syringae* pathovars are listed in Table 6. Trigonelline is a key substrate that differentiates *P*. *syringae* pv. *coronafaciens* from *P*. *syringae* pathovars with the latter being able to utilize the substrate.

**Table 6:** Phenotypic characteristics that distinguish *P*. *coronafaciens* from *P*. *syringae* and *P*. *syringae* pathovars. Data for reference taxa for column 3–7 were taken from Schaad et al. (2001).

Characteristic	1^a^	2^b^	3^c^	4^d^	5^e^	6^f^	7^g^	8^h^	9^i^
Mannitol	- ^x^	+	+	+	+	+	-	+	+
Erythritol	+	+	-	+	-	+^D^	-	+	-
Sorbitol	+	-	+	+	+	-	+	+	
Inositol	+	+	+	+	+	-	+	+	
Trigonelline	-	+	+	+	+	+	+	+	+
Gelatin Hydrolysis	+	+	-	+	+	+	+	+	+
Indole Reaction	-	+^D^	+^D^	+^D^	ND	-	-	-	
Nitrate Reduction	-	-	+	-	-	-	-	-	+
Esculin Hydrolysis	+	+	-	+^D^	+	+^D^	-	+	+
L-Lactate	-	+	-	-	+^D^	-	-	+	+^D^
D-Tartrate	-	+	-	-	-	-	+^D^	+	
Pectinolysis	-	-	-	+	ND	-	-	-	-
Fluorescence^y^	V	+	+	+	+	+	+	+	+
Ice-Nucleation	+	+	+^D^	+	-	-	+^D^	+	-

^a^*Pseudomonas coronafaciens* (ATCC 19608, NCPPB 600^PT^, YB 12–1, YB 09–1, YB 12–4 and YB 12–5).

^b^*P*. *syringae* pv. *aptata* (NCPPB 3539 and 13–4).

^c^*P*. *syringae* pv. *glycinea* (Psg 86–3).

^d^*P*. *syringae* pv. *lachrymans* (Psl 83–1).

^e^*P*. *syringae* pv. *maculicola* (Pma 02–1).

^f^*P*. *syringae* pv. *morsprunorum* (Psm 83–4).

^g^*P*. *syringae* pv. *phaseolicola* (Pph 83–2).

^h^*P*. *syringae* pv. *syringae* (NCPPB 281 ^PT^, 87–300, 88–306).

^i^*P*. *syringae* pv. *tomato* (84–17, 89–21).

^x^Adopted from Schaad et al. (2001); + = 80% or more positive; +^D^ = 80% or more delayed positive; V = 21–79% positive;— = 80% or more negative; ND = Not determined.

^y^Determined on King's Medium B.

^PT^Pathotype strain.

### Fatty acid analysis

The fatty acid profile of the *P*. *syringae* pv. *coronafaciens* (pathotype strain: NCPPB 600^PT^) was similar to that of onion strain (YB 12–1). The most abundant fatty acids identified were C_16 : 0_, C_16 : 1_ ɷ7c and/or C_16 : 1_ ɷ6c (summed feature 3) and C_18 : 1_ ɷ7c and/or C18 : 1 ɷ 7c (summed feature 8).

### Pathogenicity test

Seedlings (of tested hosts) inoculated with PBS did not produce symptoms at 15 DPI. One hundred percent of the seedlings of oat, rye and Italian ryegrass produced halo blight symptoms when inoculated with the strains of *P*. *syringae* pv. *coronafaciens* (NCPPB 600^PT^, ATCC 19608, Pcf 83–300) (Table 7). However, symptoms were not produced when strains of *P*. *syringae* pv. *coronafaciens* were inoculated on onion seedlings. In contrast, 100% of the onion seedlings displayed symptoms, when inoculated with the YB strains (12–1, 09–1, 12–4) or *P*. *syringae* pv. *porri* (NCPPB 3364^PT^) (Table 7). However, symptoms produced by YB strains were different (intense chlorosis in emerging leaves and severe blight in the older leaves) than those produced by *P*. *syringae* pv. *porri* (NCPPB 3364^PT^) (water-soaked necrotic lesions on younger leaves). Unlike *P*. *syringae* pv. *coronafaciens*, YB strains or *P*. *syringae* pv. *porri* (NCPPB 3364^PT^) strain did not produce any symptoms on the seedlings of oat, rye and Italian ryegrass. *P*. *syringae* pv. *syringae* strains (NCPPB 281^PT^, Pss 87–300) did not produce symptoms on any of the inoculated plants (Table 7). Subsequent bacterial isolation and re-identification for all bacterial strain-plant species inoculation combination reconfirmed the association of symptoms with typical bacterial strain inoculated.

**Table 7:** Pathogenicity test of *Pseudomonas coronafaciens*, *Pseudomonas syringae* pv. *syringae* and yellow bud strains on cereals, grasses and onion.

Strain	Rye (*Secale cereale*)	Oat (*Avena sativa*)	Italian ryegrass (*Lolium multoflorum*)	Onion (*Allium cepa*)
*P*. *coronafaciens*				
Pcf 83–300	+^a^	+	+	-^b^
ATCC 19608	+	+	+	-
NCPPB 600 ^PT^	+	+	+	-
Yellow bud (YB) strains				
YB 12–1	-	-	-	+^c^
YB 09–1	-	-	-	+
YB 12–4	-	-	-	+
*P*. *syringae* pv. *syringae* NCPPB 281 ^PT^	-	-	-	-
Pss 87–300	-	-	-	-
*P*. *coronafaciens* pv. *porri*				
NCPPB 3364 ^PT^	-	-	-	+

^a^ Halo blight symptoms were observed upon inoculation of bacterial suspension containing 1×10^8^ colony forming units (CFU)/ml.

^b^ No symptoms were observed on inoculated leaves.

^c^ Yellow bud symptoms were observed upon inoculation with 1×10^8^ colony forming units (CFU)/ml of bacterial suspension.

^PT^ Pathotype strain.

## Discussion

The taxonomy of *Pseudomonas syringae* and its pathovars has changed and been a matter of confusion for three decades [1]. Among the identified nine ‘genomospecies’ of the *P*. *syringae* complex, genomospecies 4 comprised of pathovars [pv. *coronafaciens*, pv. *atropurpurea*, pv. *striafaciens*, pv. *oryzae*, and pv. *zizaniae*, pv. *garcea*, pv. *porri* that infect small grains, grasses, leek, onion and coffee] [6,8]. An attempt was made by Schaad and Cunfer (1979) [9] to differentiate *P*. *syringae* pv. *coronafaciens*, *P*. *syringae* pv. *zea*, *P*. *syringae* pv. *atropurpurea* and *P*. *syringae* pv. *striafaciens*; however, the authors found these bacterial species/pathovars to be synonymous. The authors couldn’t differentiate these strains based on physiological, immunological, substrate utilization and host-range tests. Apart from these two studies, detailed investigation is lacking on characterization of *P*. *syringae* pv. *coronafaciens* and *P*. *syringae* pv. *syringae*. Despite being in genomospeies 4, “*Pseudomonas syringae* pv. *coronafaciens*” was not included in the Approved List of Bacterial Names and hence is not recognized as a valid species name [27]. Polyphasic approach of taxonomic classification was not adopted when species designations were made. The current study adopted a polyphasic approach to re-characterize *P*. *syringae* pv. *coronafaciens* strains (hosts: oat, rye and onion; and also a pathotype strain) and observed them to be distinct from the pathotype strains of *P*. *syringae* and *P*. *syringae* pathovars. Hence, it is recommended to elevate *P*. *syringae* pv. *coronafaciens* to a species level as *P*. *coronafaciens*. Furthermore, polyphasic approach was also used to identify an unknown bacterial pathogen that was responsible for a new disease in onion (yellow bud), to a species (*P*. *coronofaciens*).

Phylogenetic analysis based on 16S rRNA sequences indicate that strains of *P*. *syringae* pv. *coronafaciens* [NCPPB 600 ^PT^, ATCC 19608, 93–2, 83–300] and YB from onion (09–1, 12–1, 12–4, and 12–5), and the pathotype strains of *P*. *syringae* pv. porri (NCPPB 3364 ^PT^), *P*.*syringae* pv. *oryzae* (NCPPB 3683^PT^), and *P*.*syringae* pv. *garcea* (NCPPB 588^PT^) formed a clade that was distinct from other *P*. *syringae* pathovars. Sequencing and concatenation of four housekeeping gene loci *gltA*, *gap1*, *gyrB*, and *rpoD* resulted in a distinct clade that comprised of four YB strains (12–1, 09–1, 12–4, 12–5) and *P*. *syringae* pv. *coronafaciens* strains [NCPPB 600 ^PT^, ATCC 19608, 93–2, 83–300]. The pathotype strains of *P*. *syringae* pv. porri (NCPPB 3364 ^PT^), *P*.*syringae* pv. *oryzae* (NCPPB 3683^PT^), *P*.*syringae* pv. *striafaciens* (NCPPB 1898^PT^) and *P*.*syringae* pv. *garcea* (NCPPB 588^PT^) were also grouped in this clade, and were separated from other *P*. *syringae* pathovars. These results suggest that strains from *P*. *syringae* pv. *coronafaciens* clade are closely related and are different from other *P*. *syringae* pathovars. Similar observations were made by Gomila et al. (2017) [28] where the authors compared whole genomes and pan-genomes of 139 *Pseudomonas* pathovars. They observed that *P*. *syringae* pv. *coronafaciens* along with *P*. *syringae* pv. *garcea*, *P*. *syringae* pv. *oryzae*, *P*. *syringae* pv. *striafaciens*, and *P*. *syringae* pv. *porri* formed a distinct cluster different from other *P*. *syringae* pathovars [28]. Rombouts et al., (2015) [29] also demonstrated separate grouping of *P*. *syringae* pv. *garcea*, *P*. *syringae* pv. *oryzae*, *P*. *syringae* pv. *striafaciens*, and *P*. *syringae* pv. *porri* strains from the *P*. *syringae* pathovars using *rpoD* based sequencing and DNA fingerprinting by BOX-PCR. Although in above studies MLSA or 16S rRNA sequencing were not used but the conclusions derived from these independent studies were similar.

Plasmids not only govern bacterial host range, and microbial evolution but in some cases can be utilized in bacterial taxonomy. The knowledge of plasmid profile (quantity and type) may help in understanding bacterial phylogeny and taxonomy. However, sole or heavy reliance of plasmid diversity in bacterial taxonomy can be misleading as it can be easily transferred or lost [30]. Nevertheless, plasmid pCOR1 is common among the coronatine (a chlorosis producing phytotoxin) producing *Pseudomonas* sp. including *P*. *syringae* pv. *coronafaciens* and *P*. *syringae* pathovars (pvs. *atropurpurea*, *maculicola*, *glycinea* and *morsprunorum*) [16]. Further characterization of *P*. *syringae* pv. *coronafaciens* strains using pCOR1- based PCR assay resulted in a positive amplification from the YB strains along with pathotype strain of *P*. *syringae* pv. *coronafaciens* (NCPPB 600^PT^). However, *P*. *syringae* pathovars used in this study were not amplified indicating close relationship of the YB strains to *P*. *syringae* pv. *coronafaciens*. Based on *hrpZ* group specific PCR assay, it was observed that the YB strains along with *P*. *syringae* pv. *coronafaciens* strains belonged to group IV, which is distinct from *P*. *syringae* and *P*. *syringae* pathovars. These results indicate that the YB strains have a close relationship with the pathotype strain of *P*. *syringae* pv. *coronafaciens* (NCPPB 600) and also they are different from *P*. *syringae* pathovars. However, we acknowledge that PCR based assays reported above reflect mere presence/absence of gene or genes but they do not truly reflect their functionality.

Profile of effector genes (type) tend to be similar to some extent in closely related phytopathogenic bacterial species. The YB and *P*. *syringae* pv. *coronafaciens* strains possessed similar effector genes; *avrPto*, *avrD1*, *avrAE1*, *hopA1*, *hopB1*, *hopD1*, and *hopAF1* genes. In contrast, *P*. *syringae* pv. *syringae* possessed effector genes (*avrPto*, *avrD1*, *avrAE1*, *hopC1*, and *hopAN1*) whereas it lacked genes; *hopA1*, *hopB1*, *hopD1*, *hopF2*, *hopG1*, *hrpK1*, and *hopAF1*. These results suggest that the YB strains were similar to *P*. *syringae* pv. *coronafaciens* with respect to the presence of effector genes. Despite differences in effector profile between *P*. *syringae* pv. *coronafaciens* and *P*. *syringae* pv. *syringae*, we acknowledge that such differences may not truly reflect species level distinction. Further detailed investigation on determining effector profiles of multiple *P*. *syringae* pv. *coronafaciens* and *P*. *syringae* pv. *syringae* may throw some light on this perspective.

DNA-DNA hybridization values have been widely used by bacterial taxonomist for determining bacterial species especially in *Pseudomonas* [31]. In this study, DNA-DNA relatedness of YB strains when compared with the pathotype strains of *P*. *syringae* pv. *coronafaciens* (CFBP 2216^PT^ = NCPPB 600^PT^) exhibited ≥90.6% similarity. These results indicate that the YB and *P*. *syringae* pv. *coronafaciens* strains meet the criteria established for a bacterial species. Furthermore, DNA-DNA relatedness of a YB strain (12–1) and a pathotype strain of *P*. *syringae* pv. *coronafaciens* (CFBP 2216^PT^ = NCPPB 600^PT^) when compared with a pathotype strain of *P*. *syringae* pv. *syringae* (CFBP 4702^PT^ = NCPPB 281^PT^), similarity index of 36.2% and 34.2%, respectively were observed. These observations suggest that the YB and *P*. *syringae* pv. *coronafaciens* strains do not belong to the species *P*. *syringae*. Gardan et al. (1999) [8] also made similar observations where ≤45% DNA-DNA relatedness was observed when *P*. *syringae* CFBP 1392^PT^ was compared with *P*. *syringae* pv. *porri*, *P*. *syringae* pv. *garcea*, *P*. *syringae* pv. *striafaciens*, *P*. *syringae* pv. *coronafaciens*, *P*. *syringae* pv. *atropurpurea*, *P*. *syringae* pv. *oryzae*, and *P*. *syringae* pv. *zizaniae*.

DNA-DNA relatedness is a good indicator of species delineation and in some cases is better than 16S rRNA and MLSA. Moreover, DNA-DNA relatedness has been demonstrated to carry similar weight as that of whole genome sequencing [22]. Goris et al. (2007) [22] examined the quantitative relationship between DNA-DNA relatedness values and genome sequence-derived parameters, such as the average nucleotide identity (ANI) of common genes and the percentage of conserved DNA. The authors observed a close relationship between DNA-DNA relatedness values and ANI and the percentage of conserved DNA for each pair of strains. The authors recommended that cut-off point of 70% DNA-DNA relatedness values for species delineation more likely corresponds to 95% ANI and 69% conserved DNA. It would be interesting to evaluate relationships among the pathotype strains in genomospecies 1 including *P*. *syringae* pv. *syringae* and pathotype strains of genomospecies 4 including *P*. *syringae* pv. *coronafaciens* strains using genome sequence-derived parameter like ANI of common genes. Future studies should include comparative genomics of pathotype strains of genomospecies 1 and 4.

Pathovar is a bacterial classification that plant pathologist and applied plant microbiologists often use to differentiate bacterial strains based on their ability to cause infection on different plant host/hosts [5,19]. In this study, host range for the YB strains was determined on common hosts known for *P*. *syringae* pv. *coronafaciens* (oat, rye and Italian ryegrass) and also on an isolated host ‘onion’. As expected *P*. *coronafaciens* strains (NCPPB 600^PT^, ATCC 19608, Pcf 83–300) produced typical halo blight symptoms on oat, rye and Italian rye grass but did not produce any symptoms on onion. Contrastingly, the YB strains (12–1, 09–1, 12–4) and a pathotype strain of *P*. *syringae* pv. *porri* (NCPPB 3364^PT^) produced symptoms on onion but did not produce symptoms on any of the other tested hosts (oat, rye and Italian ryegrass). However, symptoms produced by YB strains were different (intense chlorosis in emerging leaves and severe blight in the older leaves) than those produced by *P*. *syringae* pv. *porri* (NCPPB 3364^PT^) (water-soaked necrotic lesions on younger leaves). These observations suggest that the YB strains, although belong to *P*. *syringae* pv. *coronafaciens* (identified in this study), did not share the common host range (oat, rye, Italian ryegrass). Also, these results indicate that the YB strains can potentially be a novel pathovar of *P*. *syringae* pv. *coronafaciens* infecting onion. This is the first report that of any *P*. *syringae* pv. *coronafaciens* infecting a member of Alliacea family (onion).

The “*P*. *syringae* pv. *coronafaciens”* strains belong to genomospecies 4 according to Garden et al. [8]. The species “*P*. *syringae* pv. *coronafaciens*” has been proposed by Schaad and Cunfer (1979) [9] based on phenotypic characteristics. Recently, whole genome and pan-genome comparison of 139 *Pseudomonas* pathovars revealed that *P*. *syringae* pv. *coronafaciens* belonged to a distinct cluster different from other *P*. *syringae* pathovars and hence the authors proposed to revive “*P*. *syringae* pv. *coronafaciens”* as a nomenspecies [28]. However, the study by Gomila et al. (2017) [28] lacked relevant information on phenotypic and genotypic characterization of *P*. *syringae* pv. *coronafaciens* strains that we provide in the current study and thereby proposing to designate and revive *P*. *coronafaciens* as a separate species.

In conclusion, the genotypic and phenotypic data presented in this study demonstrate that the *P*. *syringae* pv. *coronafaciens* strains along with the YB strains from onion belong to a separate species from *P*. *syringae*. We therefore propose to elevate *P*. *syringae* pv. *coronafaciens* to a species level as *P*. *coronafaciens* sp. nov., nom. rev.

### Description of *Pseudomonas coronafaciens* sp. nov., (co.ro.na.faʹci.ens. L. *corona* crown; L. *facio* to make; M.L. part. adj. *coronafaciens* halo-producing)

Bacterial cells are gram-negative, short rods, non-capsulated, motile and non-spore-forming. Colonies are cream-colored, smooth, round and convex with entire margins on nutrient agar supplemented with 0.5% yeast extract. Growth occurs at 24–30°C, but not at 4 or 40°C. The bacterium is strictly aerobic and negative for indole activity. It produces levan, is negative for oxidase, does not cause a rot in potato, negative for arginine dihydrolase, but produces a hypersensitive reaction in tobacco (LOPAT reaction: +—+). The bacterium is ice-nucleation positive and is variable for the production of a water-soluble, fluorescent pigment when grown on KMB. *P*. *coronafaciens* sp. nov., nom. rev. can hydrolyze aesculin and gelatin and utilize the following substrates at 24°C: tween 40, tween 80, L-arabinose, D-arabitol, I-erythritol, D-fructose, D-galactose, α-D-glucose, sucrose, methyl pyruvate, mono-methyl succinate, acetic acid, cis-aconitic acid, formic acid, D-galactonic acid lactone, D-galacturonic acid, D-gluconic acid, D-glucosaminic acid, D-glucuronic acid, α-keto-glutaric acid, malonic acid, propionic acid, quinic acid, D-saccharic acid, succinic acid, succinamic acid, glucuronamide, D-alanine, L-alanyl-glycine, L-asparagine, L-aspartic acid, L-Glutamic acid, glycyl-L-glutamic acid, L-proline, L-serine, L-thronine, γ-amino butyric acid, inosine, uridine, thymidine, glycerol, and D, L- α-glycerol phosphate. The bacterium cannot utilize trigonelline, L-lactate, and D-tartarate.

The type strain of *P*. *coronafaciens* sp. nov. is NCPPB 600^T^ = CFPB 2216 ^T^ = LMG 5060 ^T^ = ICMP 3316 ^T^. The most abundant fatty acids are C_16 : 0_, C_16 : 1_ ɷ7c and/or C_16 : 1_ ɷ6c (summed feature 3) and C_18 : 1_ ɷ7c and/or C18 : 1 ɷ 7c (summed feature 8). The DNA G+C% for *P*. *coronafaciens* type strain (NCPPB 600^PT^) and onion strain (YB 12–1) were 58.2 and 57.8 mol%, respectively. The NCBI accession number for the type strain is 16S rRNA gene is HM032070.
